# TAS2R38 polymorphisms and oral diseases in Thais: a cross-sectional study

**DOI:** 10.1186/s12903-022-02043-2

**Published:** 2022-01-28

**Authors:** Sawita Khimsuksri, Jarin Paphangkorakit, Waranuch Pitiphat, Susan Elaine Coldwell

**Affiliations:** 1 Department of Oral Biomedical Science, Faculty of Dentistry, Khon Kaen University, Khon Kaen, Thailand; 2 Department of Preventive Dentistry, Faculty of Dentistry, Khon Kaen University, Khon Kaen, Thailand; 3 Oral Health Sciences, School of Dentistry, University of Washington, Room B-509, Health Sciences Building, Box 357475, Seattle, WA 98195-7475 USA

**Keywords:** TAS2R38, Bitter, Genetics, Caries, Periodontitis

## Abstract

**Background:**

Polymorphisms at positions 49, 262, and 296 in the TAS2R38 bitter taste receptor gene result in two common genetic haplotypes, PAV and AVI, named for the resulting amino acid substitutions. TAS2R38 genotype has been previously associated with caries risk in children. This study aimed to identify TAS2R38 polymorphisms among Thais and to explore any association between genotype and oral diseases.

**Methods:**

Patients seeking care at Khon Kaen University Dental Hospital in Thailand were recruited to participate in the study. Saliva was collected for DNA extraction and genotyping. Patients completed a questionnaire to collect demographic variables and assess oral self-care behaviors. A calibrated dentist conducted an examination that included periodontal charting and recording of decayed, missing, and filled teeth (DMFT).

**Results:**

A total of 250 patients (19–75 years) were enrolled in the study (116 males). Two haplotypes, PAV (67.2%) and AVI (32.8%) were found, resulting in 3 diplotypes; PAV/PAV (46.0%), PAV/AVI (42.4%) and AVI/AVI (11.6%). DMFT and periodontal status of 238 participants were recorded. The three diplotype groups were similar in age, sex, socio-economic indicators, oral self-care, and number of teeth. The odds of having periodontal disease, defined as at least one site with probing depth ≥ 5 mm, were lower in AVI/AVI and PAV/AVI compared with PAV/PAV. PAV/AVI tended to have less DMFT, while AVI/AVI tended to have more DMFT compared with PAV/PAV, however these trends did not reach statistical significance.

**Conclusions:**

The frequency distribution of TAS2R38 genotypes was similar to that reported for other Asian populations. AVI/AVI genotype was associated with decreased prevalence of periodontal disease among Thai dental patients, whereas there was no significant association between TAS2R38 genotype and prevalence of tooth decay in this patient population.

## Background

The human T2R38 bitter taste receptor gene, TAS2R38, has variation in its nucleotide sequence that alters its function. Single nucleotide polymorphisms (SNPs) are found at the amino acid positions A49P, V262A, and I296V, resulting in two common genetic haplotypes, AVI and PAV, named for the amino acid substitutions that result from SNPs at those positions. Individuals who carry PAV/PAV and PAV/AVI genotypes are sensitive to the bitterness of phenylthiocarbamide (PTC) and propylthiouracil (PROP), whereas AVI/AVI individuals have greatly reduced ability to detect the bitterness of PTC/PROP [1, 2]. Two previous studies of PTC taste sensitivity of Thai participants, one in Bangkok [3] and the other in Chiang Mai [4], have observed a low prevalence of non-tasters, ranging from 4.6 to 9.7%. This is consistent with observations that Asian populations generally have a lower proportion of PTC/PROP non-tasters compared with European populations.

A number of genes have been implicated in caries susceptibility, including genes involved in enamel formation, salivary buffering capacity, immune response, and taste perception [5–8]. With regards to the taste system, a recent systematic review of the use of PROP taste testing as a caries risk assessment method in children concluded that non-tasters of PROP do have more decayed, missing and filled teeth [9]. However, the quality of evidence in support of the association between PROP taste sensitivity and caries experience was overall reported to be very low. Another recent meta-analysis did conclude that A49P (rs713598) is likely involved in susceptibility to dental caries [10]. Suggested pathways through which this gene might impact caries have included dietary preferences, salivary factors, tooth eruption timing, and even thyroid function [11–14].

More recently, the T2R38 protein, which mediates the perceived bitterness of PTC/PROP in the taste system, has been widely observed in non-gustatory tissues. T2R38 receptors in epithelial cells of the gastrointestinal tract have enteroendocrine effects related to control of metabolic functions that impact diabetes and obesity [15, 16]. T2R38 receptors in airway epithelial cells are involved in autocrine and paracrine functions regulating innate immune response against bacterial infection [17]. T2R38 protein is also expressed in epithelial lining cells of the airway and innate immune cells like neutrophils [18] and macrophages [19] that can be activated by gram-negative bacterial compounds. Additionally, T2R38 is expressed by gingival epithelial cells and impacts the innate host defense response of those cells in the presence of oral bacteria [20]. T2R38 receptors are thus important in modulating innate immune function, such as the host defense against bacterial biofilm formation that may lead to chronic infection and inflammation-related diseases.

Recent in vitro work has suggested that TAS2R38 genotype is important in mediating the response of gingival epithelial cells to bacteria involved in both caries and periodontal disease [20]. Gingival epithelial cells with the PAV/PAV genotype differentially upregulated T2R38 in response to the cariogenic pathogen *Streptococcus mutans*, whereas cells with the AVI/AVI genotype differentially upregulated T2R38 in the presence of the periodontal pathogen *Porphyromonas gingivalis*. It was also reported that gingival epithelial cells with AVI/AVI genotype increased release of human beta defensin 2 (hbD-2) in response to *Fusobacterium nucleatum*, another bacteria associated with periodontal disease. The hbD-2 response in the presence of *F. nucleatum* was reversed by silencing TAS2R38 gene expression [20]. Taken together, these findings suggest that the PAV form of the T2R38 receptor is more responsive to cariogenic bacteria, whereas the AVI form of the T2R38 receptor is more responsive to bacteria involved in periodontal disease.

The current study aims to examine TAS2R38 genotype frequency distribution among adult dental patients and to explore the relationships between TAS2R38 genotype and the presence of oral diseases (dental caries and periodontal disease) in the Thai population. We hypothesized that patients who have the PAV/PAV genotype would have lower dental caries prevalence, whereas those who have the AVI/AVI genotype would have lower periodontal disease prevalence.

## Methods

### Study population

This cross-sectional study was conducted among patients seeking dental care at Khon Kaen University (KKU) Dental Hospital in Khon Kaen, Thailand, from June 2017 to May 2019. Inclusion criteria were patients aged 18 years and older. Exclusion criteria were current smoker, xerostomia, diabetes mellitus, immunocompromised condition, autoimmune diseases, antibiotics use in the past three months, anti-inflammatory drugs use in the past six months, and significant cognitive or communication problems that may affect the ability to answer the questions. Participants had no symptoms of cold, flu, sinusitis, pharyngitis, salivary gland infections or other related condition during the data collection visits. The prevalence of PAV/PAV genotype was reported to be about 17% among all race/ethnicity groups and ranges between 16–22% in study subgroups [21]. We thus made a recruitment target of 250 participants to be able to detect the prevalence of 20% or lower.

This research was conducted in accordance with the Declaration of Helsinki. The study obtained ethical approval from the Institutional Review Board of the University of Washington, Seattle, USA (STUDY00002762), and KKU Ethics Committee in Human Research (HE592279). All participants provided written, informed consent for participation in the study.

### Data collection

**Fig. 1:**
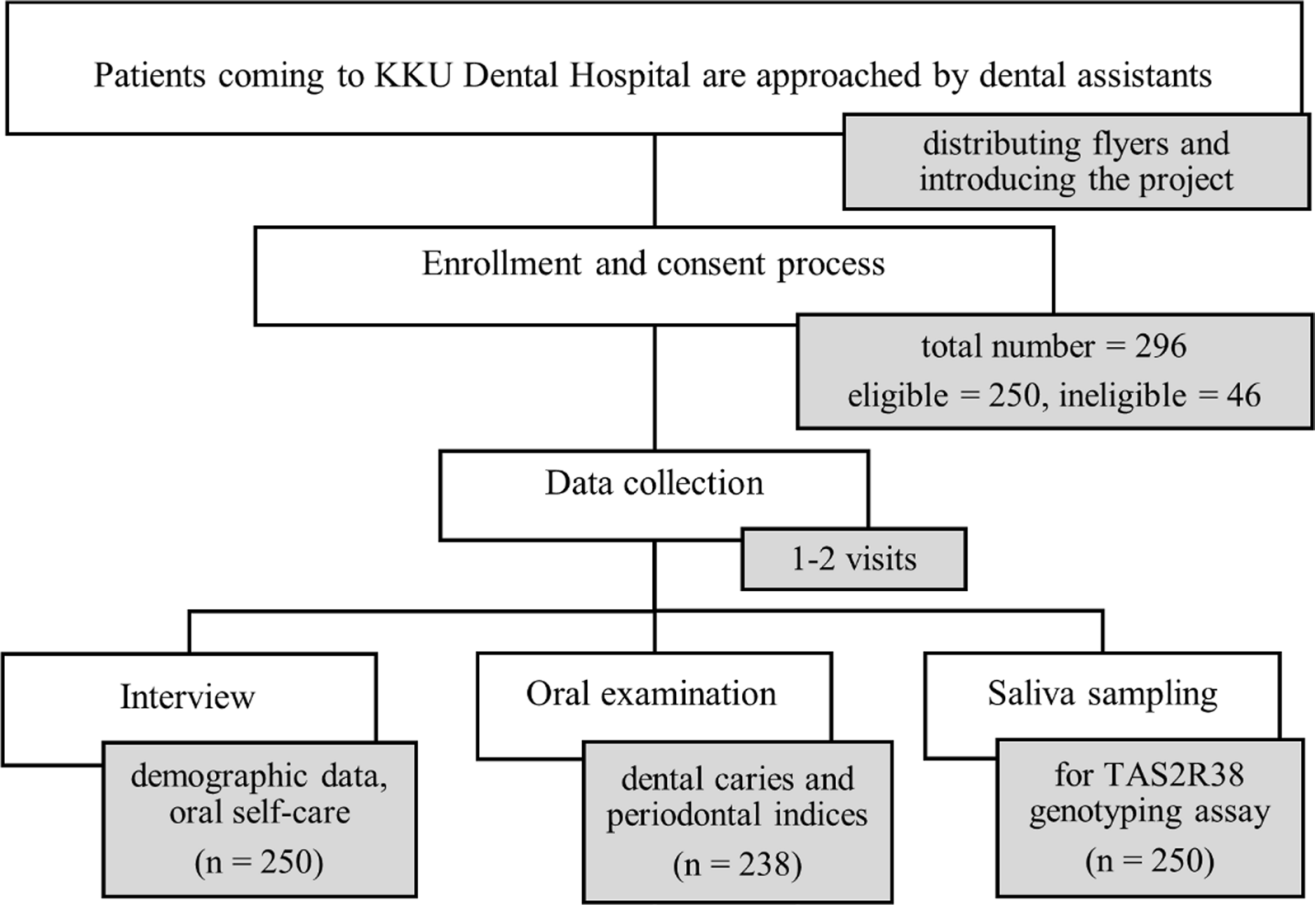
Data collection process. Flow chart describes data collection process and number of participants included in each step

Enrollment, written consent process, interview, and saliva sample collection were done at KKU Dental Hospital. Information recorded from an interview included demographic data and personal oral self-care information (Fig. 1).

One calibrated dentist performed clinical examinations on all teeth, excluding third molars, in a clinical setting. The dentist was blinded to patient genotype at the time of the examination. Dental caries was determined according to the World Health Organization criteria and decayed, missing, and filled teeth (DMFT) index was calculated [22–24]. For periodontal examination, the researcher had been trained by a certified periodontist over 4 clinical sessions to achieve ≥ 80% agreement, also any difference was ≤ 1 millimetre (mm). We used a CP-15 periodontal probe to measure and record periodontal measures in 6 locations around each tooth, including probing depth (PD) and gingival margin position (GM) in mm. Clinical attachment loss (CAL) was calculated using PD and GM.

### Saliva collection

We used Oragene-DNA kits (OG-500, DNA Genotek Inc**.**, ON, Canada) to collect 2 ml of whole saliva samples by following the manufacturer’s instructions. Samples were mailed to Monell Chemical Senses Center, PA, USA for TAS2R38 genotyping assays.

### TAS2R38 genotyping assay

DNA extraction was done following the protocol for samples collected with the OG-500 kits, using prepIT®•L2P (PT-L2P) (prepIT® L2P, DNA Genoteck Inc., ON, Canada). Genetic variation of TAS2R38 (NCBI Reference Sequence: NM_176817.5) was explored at 3 SNPs: rs713598 (C/G), rs1726866 (G/A), and rs10246939 (C/T) (C___8876467_10, C___9506827_10, and C___9506826_10, respectively; TaqMan®, ThermoFisher Scientific, CA, USA), using real-time polymerase chain reaction (PCR) single nucleotide polymorphism assays [25, 26]. Then, haplotypes and diplotypes were identified and recorded, using Applied Biosystem™ StepOnePlus® Real-Time PCR systems (Applied Biosystems® by Life Technologies™, ThermoFisher Scientific, CA, USA) for genotyping experiments. The genotyping was performed blind to the clinical status of the patients.

### Data analysis

We analyzed continuous variables as mean and standard deviation. For categorical variables, we reported counts and percentages. We used one-way ANOVA to compare means. Proportion was tested using chi-square tests. Kruskal–Wallis test was used to compare the difference among groups for skewed data and to test the proportion trend. For the disease association study, we used logistic regression, and the results were presented as odds ratios (OR) and 95% confidence interval (CI).

Participants with three common genotypes were classified according to the data from the oral examinations. Dental caries measures included mean number of decayed, missing and filled teeth due to dental caries (mean DMFT) and prevalence of having DMFT ≥ 1. Various case definitions of periodontal disease have been employed in previous studies. Therefore, we explore the association between the genotypes and periodontal disease using different cut-offs, including the prevalence and extent (% of sites) of PD ≥ 4, 5 mm and CAL ≥ 3, 4, 5 mm.

StataIC 16 (StataCorp LLC, Texas, USA) was used for data analysis. The significance level was set at 5%.

## Results

A total of 250 adult patients (age 19–75 years, mean age 30.5 years) were enrolled in this study. There were 116 (46.4%) males and 134 (53.6%) females. Most participants were in the age group of 18–24 years (44.8%) and 25–34 years (27.6%) and considered themselves as Thai (70.4%) and Chinese-Thai (24.0%). Eighty-six percent of the participants’ hometown was in Northeastern Thailand. Most of the participants completed high school and higher education, and had monthly income of ≤ 20,000 Thai Baht (Table 1).

**Table 1:** General Characteristics of study population (N = 250)

Number of males (%)	116 (46.4)
Mean age ± SD	30.5 ± 11.7
Hometown region, n (%)	
Norther	8 (3.2)
Central	19 (7.6)
Eastern	5 (2.0)
Northeastern	215 (86.0)
Southern	3 (1.2)
Race/ethnicity, n (%)	
Thai	175 (70.4)
Chinese-Thai	60 (24.0)
Other ^a^	14 (5.6)
Not known	1 (0.4)
Completed years of education, n (%)	
0–12	25 (10.0)
13–15	133 (53.2)
16+	92 (36.8)
Monthly income^b^, n (%)	
≤ 10,000	87 (36.5)
10,001–20,000	78 (32.8)
20,001–30,000	25 (10.5)
≥ 30,000	48 (20.2)

^a^Including Laos-Thai, Vietnamese-Thai, and Cambodian-Thai

^b^Presented in Thai Baht

### Frequency distribution of TAS2R38 Polymorphisms

The genetic variation at 3 sites were comprised of 2 common haplotypes: PAV (67.2%) and AVI (32.8%). These then resulted in 3 common diplotypes; PAV/PAV (46.0%), PAV/AVI (42.4%), and AVI/AVI (11.6%). The haplotype and diplotype frequency distributions were not significantly different between male and female participants. Based on 2 haplotypes, the diplotype frequency distribution was at Hardy–Weinberg equilibrium (Table 2).

**Table 2:** Genotype frequency distribution (N = 250)

TAS2R38 Genotype: n (%)				Sex		*p* value^a^
Haplotype				Male (n = 116)	Female (n = 134)	
PAV	336 (67.2)			150 (64.7)	189 (69.4)	0.26
AVI	164 (32.8)			82 (35.3)	82 (30.6)	

Diplotype		E	HWE			
PAV/PAV	115 (46.0)	113 (45.2)	0.92^a^	47 (40.5)	68 (50.7)	0.20
PAV/AVI	106 (42.4)	110 (44.0)		56 (48.3)	50 (37.3)	
AVI/AVI	29 (11.6)	27 (10.8)		13 (11.2)	16 (12.0)	

E = expected value based on 2 haplotypes

HWE = Hardy–Weinberg Equilibrium Test

^a^Chi-square test

### Association between TAS2R38 polymorphisms and oral diseases

**Fig. 2:**
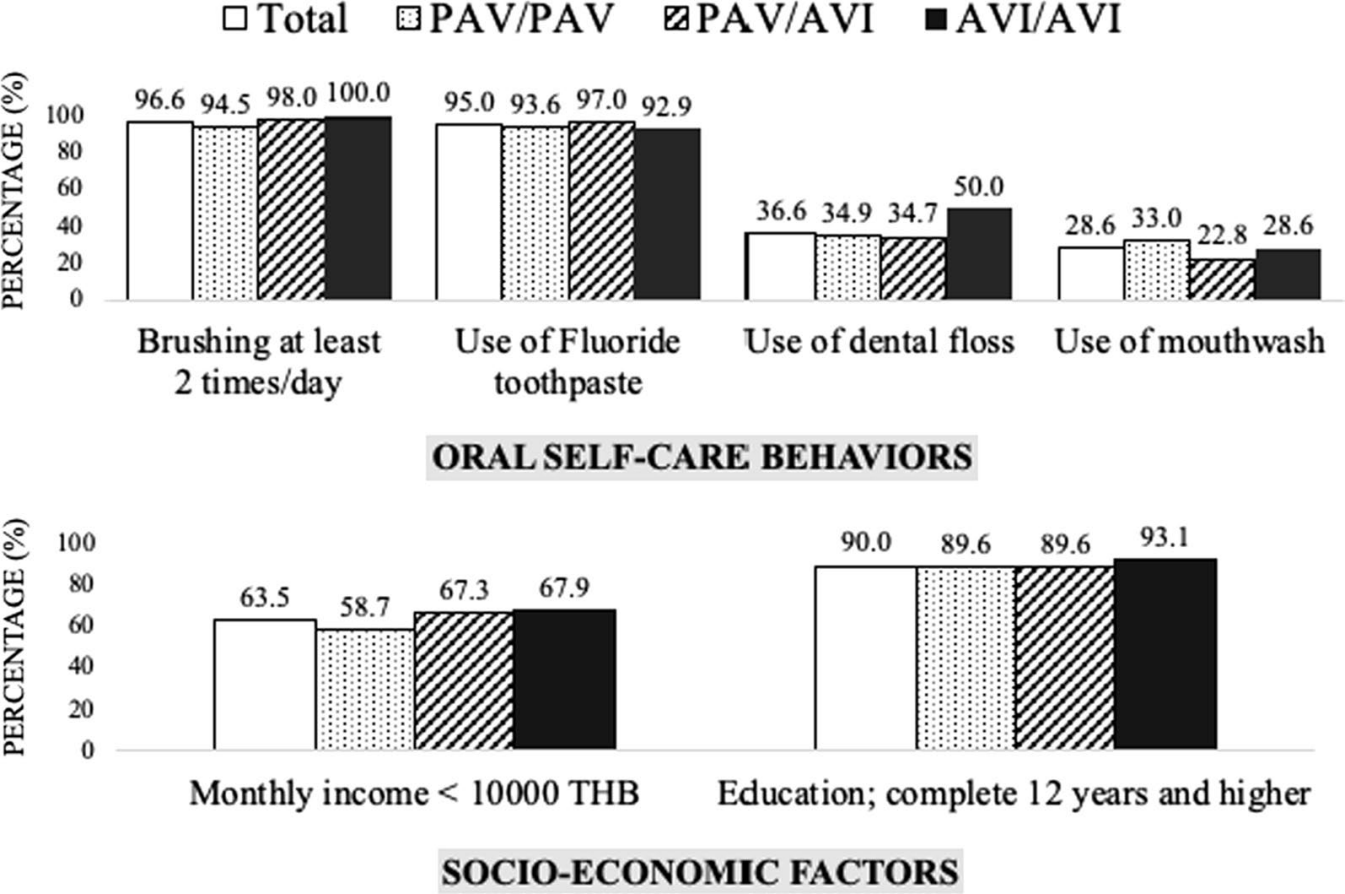
Oral self-care behaviors and socio-economic factors of study population. Oral self-care behaviors (top) and socio-economic factors (bottom) are shown for the 238 participants (white bar) and genotype subgroups (PAV/PAV: dotted, PAV/AVI: striped, and AVI/AVI: black bar) included in the portion of the study exploring the association between TAS2R38 polymorphisms and oral diseases

Twelve of the original study participants could not come back for the oral examination visit and were not included in this part of the study, leaving 238 participants. Sex proportion, average age, and the average number of teeth were not significantly different between genotype groups. Socio-economic factors and oral self-care behaviors were similar between subgroups (Fig. 2).

#### Dental caries

The average DMFT score was not significantly different among three diplotypes (p = 0.58). The AVI/AVI group tended to have higher caries experience (OR = 4.99; CI: 0.63, 39.22), while PAV/AVI tended to have lower caries experience (OR = 0.63; CI: 0.31, 1.26) when compared with PAV/PAV group. However, these differences were not statistically significant (Table 3).

**Table 3:** Association between TAS2R38 genotype and dental caries

Genotype	N	DMFT		Prevalence of dental caries (DMFT ≥ 1)	
		Mean ± SD	Range	%	OR [95% CI]^a^
Total	238	5.2 ± 4.5	0–27	82.8	
PAV/PAV	109	5.2 ± 4.0	0–18	84.4	Reference
PAV/AVI	101	5.0 ± 4.6	0–18	77.2	0.6 [0.3–1.3]
AVI/AVI	28	6.0 ± 5.8	0–27	96.4	5.0 [0.6–39.2]
*p* value^b^		0.58			
*p* for trend^c^				0.61	

^a^Logistic regression

^b^One-way ANOVA

^c^Kruskal–Wallis test

#### Periodontal disease

The average PD was not significantly different among three diplotypes (2.3 ± 0.2 mm for PAV/PAV, 2.3 ± 0.2 mm for PAV/AVI and 2.3 ± 0.1 mm for AVI/AVI). However, a significant difference in the prevalence of PD ≥ 5 mm was found among groups. The odds of having PD ≥ 5 mm was lower in AVI/AVI (OR = 0.3; CI: 0.1, 0.9) and PAV/AVI (OR = 0.6; CI: 0.3, 1.1) than PAV/PAV group (p for trend = 0.02). Similar trend was also found when considering the extent of periodontal disease. The PAV/PAV group had the highest percentage of sites with PD ≥ 4 and 5 mm, followed by PAV/AVI, and AVI/AVI, although the results were not significant (Table 4).

**Table 4:** Association between TAS2R38 genotypes and periodontal disease

Prevalence	TAS2R38 Genotype (N = 238)						*p* for trend^b^
	PAV/PAV (n = 109)		PAV/AVI (n = 101)		AVI/AVI (n = 28)		
	%	OR [95%CI]^a^	%	OR [95%CI]	%	OR [95%CI]	
PD							
≥ 4 mm	86.2	Reference	70.3	0.4 [0.2–0.8]	82.1	0.7 [0.2–2.2]	0.10
≥ 5 mm	35.8	Reference	25.7	0.6 [0.3–1.1]	14.3	0.3 [0.1–0.9]	0.02*
CAL							
≥ 3 mm	89.9	Reference	80.2	0.5 [0.2–1.0]	82.1	0.5 [0.2–1.6]	0.10
≥ 4 mm	38.5	Reference	36.6	0.9 [0.3–1.6]	25.0	0.5 [0.2–1.4]	0.26
≥ 5 mm	19.3	Reference	13.9	0.7 [0.3–1.4]	7.1	0.3 [0.1–1.5]	0.09

Extent (% sites)	Mean ± SD		Mean ± SD		Mean ± SD		*p* value^b^
PD							
≥ 4 mm	4.7 ± 6.5		3.8 ± 5.6		3.2 ± 3.7		0.14
≥ 5 mm	0.7 ± 2.1		0.6 ± 1.7		0.3 ± 1.0		0.09
CAL							
≥ 3 mm	9.4 ± 16.5		8.1 ± 12.6		6.7 ± 11.5		0.81
≥ 4 mm	3.1 ± 9.5		2.2 ± 6.9		1.5 ± 5.0		0.42
≥ 5 mm	1.2 ± 4.4		0.8 ± 3.3		0.6 ± 2.6		0.27

*PD* probing depth, *CAL* clinical attachment loss, *SD* standard deviation

*Statistically significant trend

^a^Logistic regression

^b^Kruskal–Wallis test

The average CAL was not significantly different between 3 diplotypes (1.4 ± 0.6 mm for PAV/PAV, 1.3 ± 0.6 mm for PAV/AVI and 1.3 ± 0.5 mm for AVI/AVI). There were some non-significant trends in the expected direction. The AVI/AVI group tended to have lowest percentage of sites with CAL ≥ 3, 4, 5 mm, while PAV/PAV tended to have the highest. A nearly significant trend (p for trend = 0.09) of the prevalence was observed in CAL ≥ 5 mm (Table 4).

## Discussion

The present study has reported TAS2R38 polymorphisms in Thais. The distribution of genotypes for TAS2R38 in a sample of dental patients from Northeastern Thailand was similar to the distribution of genotypes observed for other Asian populations studied. The proportion of participants with AVI/AVI diplotype was low (11.6%) compared with populations of European origin [21, 27]. This result is in agreement with the low prevalence of non-tasters of PTC previously reported for Thais [3, 4]. The genotypes differed from sub-Saharan African and North American populations previously studied in that only the two most common haplotypes (PAV, AVI) were observed in our samples [27]. Our results were comparable to those of Malaysians reported by Ooi et al. [28]. The majority of the Malaysian subjects were Malay and Chinese, in which the Chinese ethnic group carried the most PAV haplotype. There were 60 participants (24%) in our study who considered themselves as Chinese-Thai. Their genotype frequency distribution was similar to those who considered themselves as Thai, suggesting a close ethnic relationship between the two [4]. In contrast, rare haplotypes (AAV, AAI) have been reported in Caucasians and Africans, resulting in different genotypes among those populations [21, 27]. In many Asian populations, high sensitivity to PTC/PROP is much more common, while non-tasters are less common than in populations of Caucasian and African descent. Similarly, the non-taster genotype was observed to be relatively rare in our sample. Nevertheless, the diplotypes were in Hardy–Weinberg Equilibrium. It was therefore appropriate to use these genotype results to further study the association between TAS2R38 genotypes and oral diseases.

Previous work has suggested that the PAV haplotype of TAS2R38 confers protection against dental caries in primary dentition [14]. Children who are PROP tasters have also been found to have lower dental caries experience in their permanent teeth [29, 30]. We observed some trends for reduced risk of dental caries in permanent dentition, although these results were not statistically significant. The lack of association of TAS2R38 genotype with caries in these adult patients is consistent with prior studies, which also did not observe an association of caries with PTC/PROP tasting and TAS2R38 genotype in adults [11, 14]. One potential explanation is that the PAV form of T2R38 may be of most benefit in protecting against ‘early colonization’ of the oral cavity by cariogenic pathogens [29, 30].

A previous in vitro study found that gingival epithelial cells with the AVI/AVI genotype increased expression of T2R38 by 4.4 folds when stimulated with *P. gingivalis* [20]. Additionally, AVI/AVI gingival epithelial cells (but not PAV/PAV cells) increased secretion of hbD-2 in response to stimulation with *F. nucleatum*. This increase was reversed by silencing T2R38 expression [20]. A recently published series of studies using a mouse model demonstrated that gingival solitary chemosensory cells were involved in innate immune response in periodontitis, and that taste receptors were involved in mediating this response [31]. These laboratory studies suggest that taste receptors in the gingiva are important in modulating the host response to periodontal pathogens and likely play a role in susceptibility to periodontal disease.

To our knowledge, this is the first report of the relationship between TAS2R38 genotype and periodontal disease. PD and CAL are commonly used in assessing periodontitis [32, 33]. We did not find a significant association between genotype at any levels of CAL, in terms of both the prevalence and extent. Because most participants were young adults who had low levels of periodontal tissue destruction, CAL may not be a sensitive measure of periodontal disease in the present study. On the other hand, there was a significant association of genotype and the prevalence of PD ≥ 5 mm, suggesting that AVI was protective against periodontal disease. Having at least one site of PD ≥ 5 mm is defined as mild periodontitis based on the American Academy of Periodontology/Centers for Disease Control and Prevention (AAP/CDC) case definitions [32].

We approached all patients coming to KKU dental hospital. Age distribution and sex proportion of this study population were similar to that of Northeastern Thai, and Thai population [34]. Oral self-care behaviors of the study population were comparable to that previously reported for the Thai population [35]. Only healthy adults with no history of current smoking were included to control for other risk factors. The three genotype subgroups were similar in general characteristics, socio-economic factors and oral self-care behaviors. This indicates that the trend and difference found in the present study were likely to be due to the difference in genotypes. The average education level and monthly income were higher when compared to general Thais but these factors were not associated with genotype. Taken together, these characteristics indicate that our sample is similar to Northeastern Thai and Thai population and our genotype frequency distribution could be generalized to Northeastern Thais and Thais.

The present study had some limitations. Firstly, we did not base the sample size of the study on power required for the genotype and oral disease association study. Therefore, these exploratory parts might not have enough power to detect the association, if one exists. Secondly, the use of convenience sampling in the present study may limit generalizability of the findings. However, the general characteristics which were related to TAS2R38 polymorphisms of the participants were similar to those of general Thais. Thirdly, with this cross-sectional study design, the data are not suitable to be used for causal inference. Thus, this study cannot be used to conclude that specific TAS2R38 genotypes cause or prevent oral diseases, but rather may be risk indicators to be considered in adult Thai patients. Lastly, although the genotyping assays were done with triplicate experiments for internal control, additional independent replication was not performed as an external control. The present study has shown the association between AVI/AVI genotype and reduced prevalence of periodontal disease. Taste phenotyping for dental patients could therefore be useful in the prediction of their susceptibility to future periodontal breakdown. However, future research will be needed to confirm the association between clinical signs of periodontal disease and TAS2R38 genotype observed in the current study. Case–control studies could be used to ensure that adequate numbers of patients exhibiting clear clinical evidence of periodontal disease are included. Another approach would be to increase the number of participants with AVI/AVI genotype in order to increase the study’s power to detect the genotype and oral disease association.

## Conclusion

TAS2R38 genotype frequency distribution in Thai dental patients is similar to other Asian populations. AVI/AVI genotype was associated with decreased prevalence of periodontal disease, whereas there was no association between TAS2R38 genotype and dental caries in Thai adults in this study. Should these trends be confirmed, TAS2R38 phenotyping could be beneficial in assessing disease prognosis. Also, T2R38 could be considered as a specific target for adjunctive treatments by activating innate immune response using bitter compound.

## Data Availability

The datasets collected and analyzed during the current study are available from the first author on reasonable request.

## Abbreviations

AAI: Alanine-Alanine-Isoleucine, rare haplotype of the TAS2R38 bitter taste receptor gene
AAV: Alanine-Alanine-Valine, rare haplotype of the TAS2R38 bitter taste receptor gene
AVI: Alanine-Valine-Isoleucine, non-taster haplotype of the TAS2R38 bitter taste receptor gene
°C: Degree celsius
CAL: Clinical attachment loss
CI: Confidence interval
DMFT: Dental caries index—decayed, missing, and filled tooth
hBD-2: Human antimicrobial peptide beta-defensin 2
mm: Millimeter
OR: Odds ratio
PAV: Proline–Alanine–Valine, taster haplotype of the bitter TAS2R38 bitter taste receptor gene
PCR: Polymerase chain reaction
PD: Probing depth
PROP: Propylthiouracil
PTC: Phenylthiocarbamide
SNPs: Single nucleotide polymorphisms
T2R38: Bitter taste receptor type 2 member 38
TAS2R38: Human T2R38 bitter taste receptor gene

## References

[1] Kim UK, Jorgenson E, Coon H, Leppert M, Risch N, Drayna D (2003). Positional cloning of the human quantitative trait locus underlying taste sensitivity to phenylthiocarbamide. Science.

[2] Drayna D (2005). Human taste genetics. Annu Rev Genom Hum Genet.

[3] Simmons RT, Graydon JJ, Sringam S (1954). A blood group genetical survey in Thais, Bangkok. Am J Phys Anthropol.

[4] Boobphanirojana P, Chetanasilpin M, Saengudom C, Flatz G (1970). Phenylthiocarbamide taste thresholds in the population of Thailand. Humangenetik.

[5] Vieira AR, Modesto A, Marazita ML (2014). Caries: review of human genetics research. Caries Res.

[6] Yildiz G, Ermis RB, Calapoglu NS, Celik EU, Turel GY (2016). Gene-environment Interactions in the Etiology of Dental Caries. J Dent Res.

[7] Chisini LA, Cademartori MG, Conde MCM, Costa FDS, Tovo-Rodrigues L, Carvalho RV (2020). Genes and SNPs in the pathway of immune response and caries risk: a systematic review and meta-analysis. Biofouling.

[8] Chisini LA, Cademartori MG, Conde MCM, Tovo-Rodrigues L, Correa MB (2020). Genes in the pathway of tooth mineral tissues and dental caries risk: a systematic review and meta-analysis. Clin Oral Investig.

[9] Alkuhl H, Morgan R, Koletsi D, Kavvadia K (2021). Genetic taste sensitivity and dental caries in children and adolescents: a systematic review and meta-analysis. Int J Paediatr Dent.

[10] Chisini LA, Cademartori MG, Conde MCM, Costa FDS, Salvi LC, Tovo-Rodrigues L (2021). Single nucleotide polymorphisms of taste genes and caries: a systematic review and meta-analysis. Acta Odontol Scand.

[11] Chung CS, Witkop CJ, Henry JL (1964). A genetic study of dental caries with special preference to PTC taste sensitivity. Am J Hum Genet.

[12] Chung CS, Witkop CJ, Wolf RO, Brown KS (1965). Dental caries in relation to PTC taste sensitivity, secretor status, and salivary thiocyanate level. Arch Oral Biol.

[13] Lin BP (2003). Caries experience in children with various genetic sensitivity levels to the bitter taste of 6-n-propylthiouracil (PROP): a pilot study. Pediatr Dent.

[14] Wendell S, Wang X, Brown M, Cooper ME, DeSensi RS, Weyant RJ (2010). Taste genes associated with dental caries. J Dent Res.

[15] Latorre R, Huynh J, Mazzoni M, Gupta A, Bonora E, Clavenzani P (2016). Expression of the bitter taste receptor, T2R38, in enteroendocrine cells of the colonic mucosa of overweight/obese vs. lean subjects. PLoS ONE.

[16] Pham H, Hui H, Morvaridi S, Cai J, Zhang S, Tan J (2016). A bitter pill for type 2 diabetes? The activation of bitter taste receptor TAS2R38 can stimulate GLP-1 release from enteroendocrine L-cells. Biochem Biophys Res Commun.

[17] Cohen NA (2017). The genetics of the bitter taste receptor T2R38 in upper airway innate immunity and implications for chronic rhinosinusitis. Laryngoscope.

[18] Maurer S, Wabnitz GH, Kahle NA, Stegmaier S, Prior B, Giese T (2015). Tasting *Pseudomonas aeruginosa* biofilms: human neutrophils express the bitter receptor T2R38 as sensor for the quorum sensing molecule N-(3-oxododecanoyl)-l-homoserine lactone. Front Immunol.

[19] Gaida MM, Dapunt U, Hansch GM (2016). Sensing developing biofilms: the bitter receptor T2R38 on myeloid cells. Pathog Dis.

[20] Gil S, Coldwell S, Drury JL, Arroyo F, Phi T, Saadat S (2015). Genotype-specific regulation of oral innate immunity by T2R38 taste receptor. Mol Immunol.

[21] Mennella JA, Pepino MY, Duke FF, Reed DR (2010). Age modifies the genotype-phenotype relationship for the bitter receptor TAS2R38. BMC Genet.

[22] World Health Organization (2013). Oral health surveys: basic methods.

[23] Idrees M, Hammad M, Faden A, Kujan O (2017). Influence of body mass index on severity of dental caries: cross-sectional study in healthy adults. Ann Saudi Med.

[24] Manpreet K, Ajmal MB, Raheel SA, Saleem MC, Mubeen K, Gaballah K (2021). Oral health status among transgender young adults: a cross-sectional study. BMC Oral Health.

[25] Bufe B, Breslin PA, Kuhn C, Reed DR, Tharp CD, Slack JP (2005). The molecular basis of individual differences in phenylthiocarbamide and propylthiouracil bitterness perception. Curr Biol.

[26] Douglas JE, Lin C, Mansfield CJ, Arayata CJ, Cowart BJ, Spielman AI (2019). Tissue-dependent expression of bitter receptor TAS2R38 mRNA. Chem Senses.

[27] Risso DS, Mezzavilla M, Pagani L, Robino A, Morini G, Tofanelli S (2016). Global diversity in the TAS2R38 bitter taste receptor: revisiting a classic evolutionary PROPosal. Sci Rep.

[28] Ooi SX, Lee PL, Law HY, Say YH (2010). Bitter receptor gene (TAS2R38) P49A genotypes and their associations with aversion to vegetables and sweet/fat foods in Malaysian subjects. Asia Pac J Clin Nutr.

[29] Rupesh S, Nayak UA (2006). Genetic sensitivity to the bitter taste of 6-n propylthiouracil: a new risk determinant for dental caries in children. J Indian Soc Pedod Prev Dent.

[30] Öter B, Ulukapı I, Ulukapı H, Topçuoğlu N, Cıldır S (2011). The relation between 6-n-propylthiouracil sensitivity and caries activity in schoolchildren. Caries Res.

[31] Zheng X, Tizzano M, Redding K, He J, Peng X, Jiang P (2019). Gingival solitary chemosensory cells are immune sentinels for periodontitis. Nat Commun.

[32] Eke PI, Page RC, Wei L, Thornton-Evans G, Genco RJ (2012). Update of the case definitions for population-based surveillance of periodontitis. J Periodontol.

[33] Tonetti MS, Greenwell H, Kornman KS (2018). Staging and grading of periodontitis: framework and proposal of a new classification and case definition. J Clin Periodontol.

[34] National Statistical Office of Thailand (NSO). Number of population from registration by age group and region: 2011–2020. http://statbbi.nso.go.th/staticreport/page/sector/en/01.aspx. Accessed 8 June 2020.

[35] Bureau of Dental Health, Department of Health, Ministry of Public Health, Thailand. The 8th National Oral Health Survey. 2017. http://dental2.anamai.moph.go.th/more_news.php?cid=273&filename=Surveillance. Accessed 8 June 2020.

